# Supplementary material for: Long-Chain Polyisoprenoids Are Synthesized by AtCPT1 in Arabidopsis thaliana
Source: Molecules. 2019 Jul 31;24(15):2789. doi: 10.3390/molecules24152789 (PMC6695881; doi:10.3390/molecules24152789)
Supplement: Supplementary file 1 [file molecules-24-02789-s001.zip › Supplemental Figure S1.pdf]

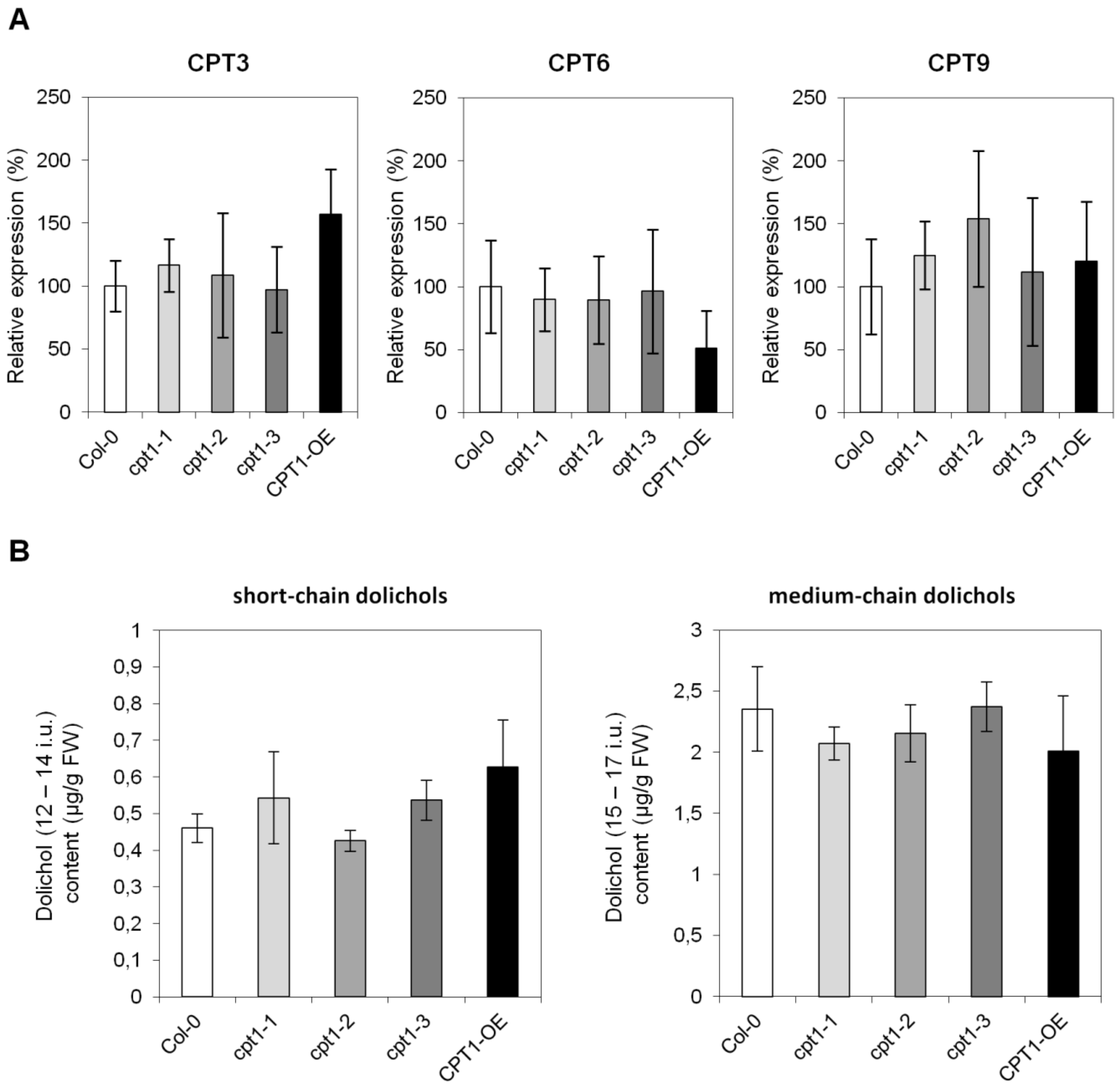

Supplemental Figure S1. (A) Expression of the other Arabidopsis CPTs and (B) content of short- and medium-chain dolichols in roots of wild type (Col-0), three lines of T-DNA insertion mutants (*cpt1-1*, *cpt1-2*, *cpt1-3*), and *AtCPT1* overexpressing (*CPT1-OE*) plants.
