# Supplementary material for: Long-Chain Polyisoprenoids Are Synthesized by AtCPT1 in Arabidopsis thaliana
Source: Molecules. 2019 Jul 31;24(15):2789. doi: 10.3390/molecules24152789 (PMC6695881; doi:10.3390/molecules24152789)
Supplement: Supplementary file 1 [file molecules-24-02789-s001.zip › Supplemental Figure S2.pdf]

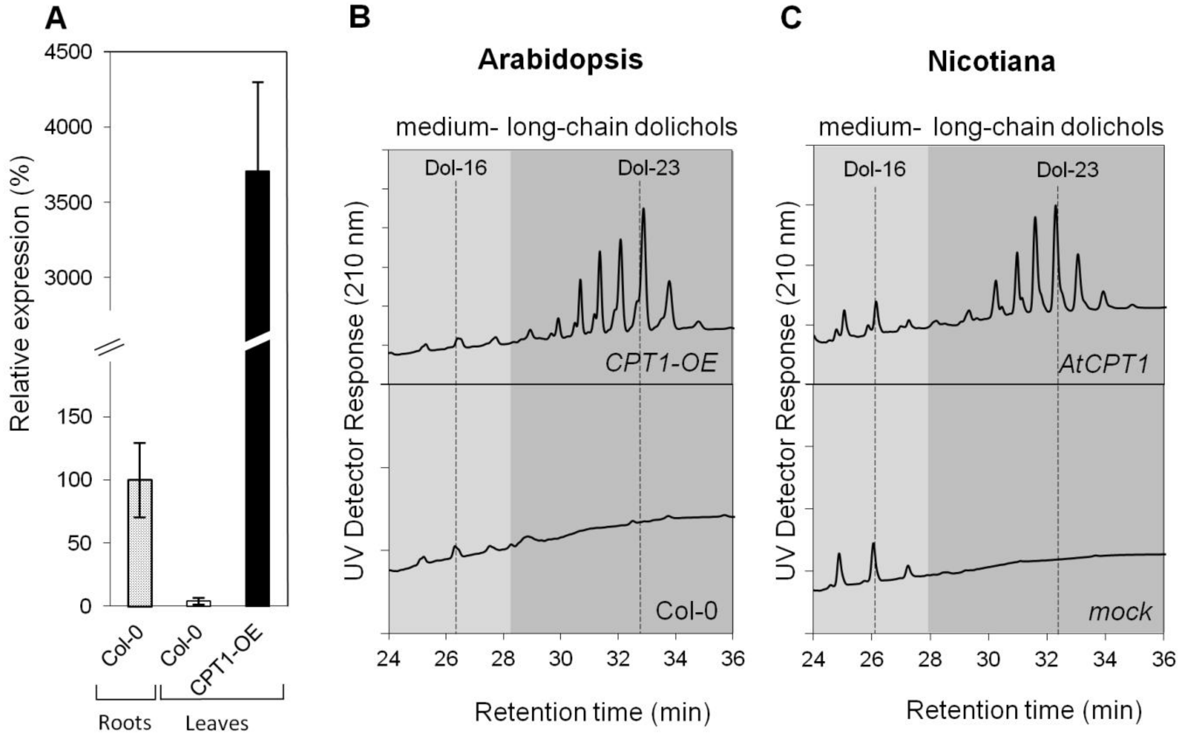

Supplemental Figure S2. *AtCPT1* overexpression leads to long-chain dolichols accumulation in Arabidopsis and Nicotiana leaves. (A) Relative expression of *AtCPT1* in Arabidopsis leaves of the wild type (Col-0) and *AtCPT1* overexpressing line (*CPT1-OE*). AtCPT1 mRNA abundance was quantified by Real-Time PCR and is presented as a percentage of the wild type roots (100%). (B) HPLC/UV chromatogram of total polyisoprenoid mixture extracted from WT and *CPT1-OE* Arabidopsis leaves. (C) HPLC/UV chromatogram of total polyisoprenoid mixture extracted from *Nicotiana benthamiana* mock leaves and those infiltrated with Arabidopsis *CPT1*, four days post infiltration (dpi). The family of dolichols (D18 – P25, with dominating P23) synthesized by AtCPT1 is indicated.
