# Supplementary material for: Long-Chain Polyisoprenoids Are Synthesized by AtCPT1 in Arabidopsis thaliana
Source: Molecules. 2019 Jul 31;24(15):2789. doi: 10.3390/molecules24152789 (PMC6695881; doi:10.3390/molecules24152789)
Supplement: Supplementary file 1 [file molecules-24-02789-s001.zip › Supplemental Table S1.pdf]

Supplemental Table S1. Synthetic oligonucleotides used in this study.

Start codons are in green, stop codons in red. Restriction sites are underlined. A description of what each primer was used for is provided.

Primer name	*Primer sequence (5'-3')*	*Description*
**Cloning into pENTR and ImpGWB vectors**		
CPT1F-5'	CACCATGTTGTCTCTTCTTTCTTCTGA	5' primer to cloning AtCPT1 into PENTR and ImpGWB 402 vectors
CPT1OER-3'	TCAAACCCGACAACCAAATC	3' primer to cloning AtCPT1 into pENTR and ImpGWB 402 vectors
CPT1GFPR-3'	ACCCGACAACCAAATCGTC	3' primer to cloning AtCPT1 into pENTR and ImpGWB 451 vectors
**Real-time PCR Analysis of AtCPT1 and other AtCPT family members**		
CPT1F-5'	GTGGCAACTTGCTTATTCCG	5' primer for *AtCPT1* expression
CPT1R-3'	CCTACGCTGATACGAAGC	3' primer for *AtCPT1* expression
CPT3F-5'	GCGCTTATGTCGATGCTG	5' primer for *AtCPT3* expression
CPT3R-3'	CAGACTCAACCTCCTCAGG	3' primer for *AtCPT3* expression
CPT6F-5'	GACGATTATGACAACGAGCAAC	5' primer for *AtCPT6* expression
CPT6R-3'	ATGTCTTGGCATCAGCTCTC	3' primer for *AtCPT6* expression
CPT9F-5'	AGCATGTGGCGGTTATATTGG	5' primer for *AtCPT9* expression
CPT9R-3'	TCTCCATGAGCCTTCTTCG	3' primer for *AtCPT9* expression
PP2AA3F-5'	TAACGTGGCCAAAATGATGC	5' primer for housekeeping gene *At*PP2AA3 expression
PP2AA3R-3'	GTTCTCCACAACCGCTTGGT	3' primer for housekeeping gene *At*PP2AA3 expression
**Genotyping of transgenic Arabidopsis plants**		
038151LP	CGTGTCTCGGCATTAGTTCTC	5' primer to AtCPT1 for *cpt1-1* mutant genotyping
038151RP	CCAAAGAGTGTCACGAGGAAG	3' primer to AtCPT1 for *cpt1-1* mutant genotyping
032276LP	CGGACTCATCAAAATCAAAGC	5' primer to AtCPT1 for *cpt1-2* mutant genotyping
032276RP	GGAGAGATTCAGGGATCTTCG	3' primer to AtCPT1 for *cpt1-2* mutant genotyping
100795LP	TGGGGGGTTCATACAGTCTCAG	5' primer to AtCPT1 for cpt1-3 mutant genotyping
100795RP	TTCGTGACGTGTCACTCTCTG	3' primer to AtCPT1 for cpt1-3 mutant genotyping
LBb1.3	ATTTTGCCGATTTCGGAAC	3' primer to T-DNA insert for cpt1 mutants genotyping
35SF-5'	CAATCCCACTATCCTTCGC	5' primer to CaMV35S promoter for CPT1-OE mutant genotyping
CPT1R-3'	CCTACGCTGATACGAAGC	3' primer to AtCPT1 for CPT1-OE mutant genotyping
**Functional complementation of the yeast CPT mutants**		
CPT1F-5'	CACCATGTTGTCTCTTCTTTCTTCTGA	5' primer to cloning AtCPT1 into pENTR and pYES-DEST52 vectors
CPT1R-3'	TCAAACCCGACAACCAAATC	3' primer to cloning AtCPT1 into pENTR and pYES-DEST52 vectors
pESC-CPT1F-5'	ATCGCGGCCGCAATGTTGTCTCTTCTTTCT	5' primer to cloning AtCPT1 into pESC-URA vector
pESC-CPT1R-3'	TAGACTAGTTCAAACCCGACAACCAAATC	3' primer to cloning AtCPT1 into pESC-URA vector
pESC-LEW1F-5'	TAGAGGATCCTATGGATTCGAATCAATCGATGC	5' primer to cloning LEW1 into pESC-URA vector
pESC-LEW1R-3'	CGTTGTCGACTTAAGTTCCATAGTTTTGGTGG	3' primer to cloning LEW1 into pESC-URA vector
